# Ultrasound of the uterosacral ligaments: A reliability study for diagnosing endometriosis in Australian non‐specialised medical imaging and radiology settings

**DOI:** 10.1002/ajum.12420

**Published:** 2025-02-18

**Authors:** Shae Maple, Eva Bezak, K Jane Chalmers, Nayana Parange

**Affiliations:** ^1^ Allied Health and Human Performance University of South Australia Adelaide South Australia Australia

**Keywords:** deep infiltrative endometriosis, gynaecology, sonographer, sonography, transvaginal ultrasound, uterosacral ligaments

## Abstract

**Introduction/Background:**

Uterosacral ligaments (USL) are the most common sites of endometriosis. Transvaginal ultrasound (TVS) demonstrates high diagnostic accuracy for endometriosis of the USLs using standardised technique and characterisations. While high accuracy and reproducibility is established with these techniques by well‐trained professionals, the question still remains if these techniques are reproducible in general settings. This study aims to assess the intra and interobserver agreement of TVS characteristics of USLs, between experienced and less experienced examiners in an Australian general ultrasound imaging practice, where sonographers are required to perform ultrasound for endometriosis.

**Methods:**

Forty‐two patients, with and without known endometriosis, underwent ultrasound imaging of the USLs. Images were obtained of uterosacral ligaments and collated for interobserver survey. Six professional observers independently reviewed the images, classifying characteristics such as echogenicity, echotexture, contour, thickness, and presence of nodules. Interobserver reliability was assessed using Gwet's agreement coefficients (*AC1*), and the correlation between USL thickness and endometriosis was analyzed using Spearman's correlation.

**Results:**

Interobserver agreement for detecting USL endometriosis was substantial (*AC1* = 0.63), with an overall agreement of (0.65) for the seven USL characteristics. Intraobserver agreement ranged from moderate (0.60) to almost perfect (0.96). Experience did not significantly affect intraobserver reliability. A strong positive correlation was found between USL thickness and endometriosis (r = 0.7965, P < 0.01).

**Conclusion:**

This study demonstrates high inter and intraobserver agreement among sonographers and radiologists in a general imaging department for identifying USL characteristics and diagnosing USL endometriosis. Both experienced and less experienced operators can reliably assess USLs Consistency was shown in identifying thickened uterosacral ligaments however, there is no consensus on where uterosacral ligament be measured. Even so, a thickened USL can prompt further extension of the pelvic scan to look for other endometriosis markers.

## Introduction

Endometriosis is a common and painful gynaecological condition affecting approximately 10% of female‐born individuals,^1^ characterised by endometrial‐like tissue growing outside the uterine cavity.^2^ This tissue can cause a range of symptoms, including menstrual pain, chronic pelvic pain and infertility.^3^, ^4^ In Australia, the average delay in diagnosing endometriosis is currently 6.4 years.^5^

**Figure 1:**
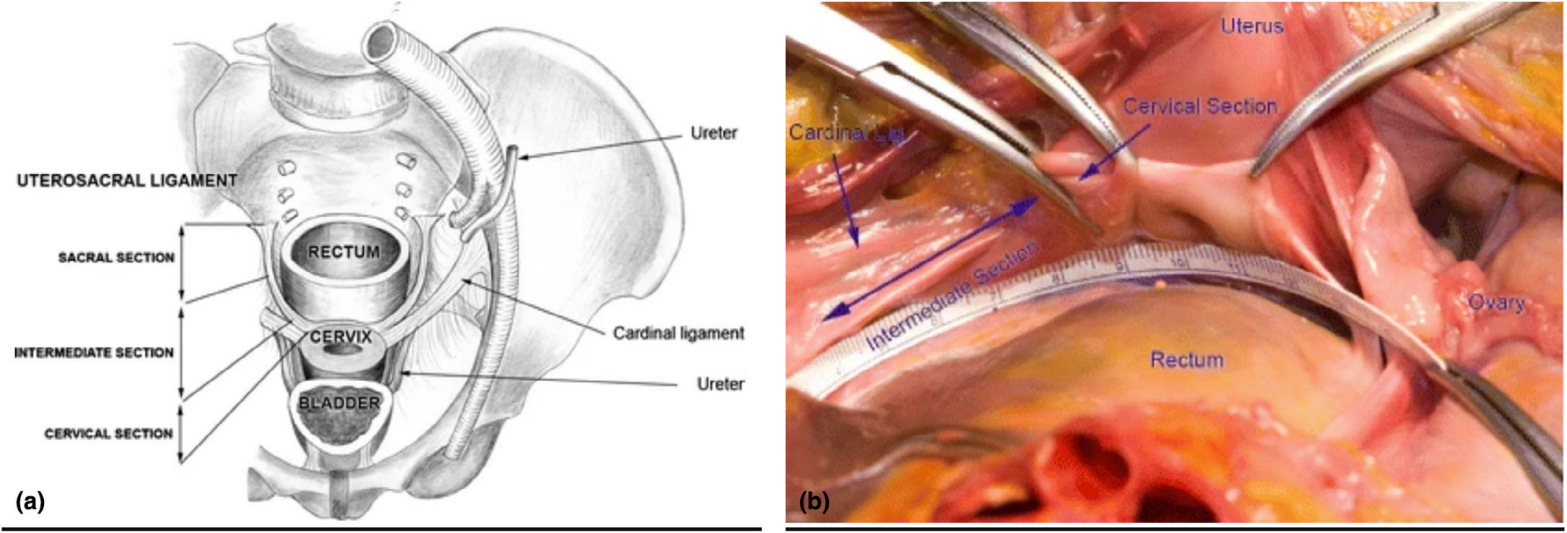
A schematic diagram of the pelvis viewed from above showing the three parts of the uterosacral ligament and its relations with the cardinal ligament near the cervix (a) and, surgical image of the USL demonstrating the three sections and surrounding anatomy (b). The blue arrows are pointing to the corresponding anatomy as labelled. Courtesy: Vu 2010.^8^ USL, uterosacral ligaments.

Multiple studies have identified the uterosacral ligaments (USL) as one of the most common sites for deep endometriosis (DE).^3^, ^4^, ^6^, ^7^ The USLs are extraperitoneal structures made of connective tissue that form the lateral boundaries of the rectouterine and rectovaginal spaces.^3^ Anatomically, the USL is divided into three sections: distal (cervical), intermediate and proximal (sacral).^8^ The distal section, which attaches to the posterior cervix, is the most easily visualised sonographically^4^, ^8^, ^9^ (Figure 1).

Historically, laparoscopy with histological examination was considered the gold standard for diagnosing endometriosis.^10^, ^11^, ^12^ However, advances in the ability and accuracy of transvaginal ultrasound (TVS) have made it the recommended first‐line diagnostic tool for endometriosis,^2^, ^9^, ^13^, ^14^, ^15^, ^16^, ^17^, ^18^, ^19^, ^20^, ^21^ helping reduce delays in diagnosis and improve surgical planning.^10^ As a result, clear and reproducible ultrasound criteria are becoming increasingly important for diagnosing DE.^10^ The International Deep Endometriosis Analysis (IDEA)^9^ consensus statement outlines the best ultrasound practices for evaluating pelvic endometriosis, with a systematic approach to assessing both the anterior and posterior compartments. While significant research has focused on endometriosis affecting the rectum and rectosigmoid, which has been well‐received within clinical practice,^9^, ^22^, ^23^ the role of USLs in the ultrasound diagnosis of endometriosis remains studied.^19^, ^24^

A recent systematic review^24^ recommended that USL examination be included in TVS examinations when DE is clinically suspected, as scanning the USLs has demonstrated clinical value in early diagnosis. However, the review also highlighted inconsistent descriptors and varying terminology used to describe USL findings and variation in ultrasound techniques.^24^ Importantly, there is currently no data on interobserver agreement regarding TVS for USL assessment.^24^ Despite this, recent studies^19^ have shown high diagnostic accuracy for assessing DE of the USLs using standardised TVS techniques.^4^, ^19^, ^24^, ^25^ Nevertheless, while it is known that TVS is a highly accurate and reproducible method for non‐invasive diagnosis of DE by well‐trained professionals, the question remains whether these techniques are reproducible in general settings.

Considering this, the aim of this study was to assess the intra‐ and interobserver agreement and reliability of TVS characteristics of normal and abnormal USLs, including echogenicity, thickness and nodules. This was done among both experienced and less experienced examiners in an Australian general ultrasound practice, where sonographers are responsible for performing ultrasounds to diagnose endometriosis.

## Methods

### Study design

This study was a prospective intra‐ and interobserver analysis conducted between November 2021 and September 2022. The methodology adhered to the recommended Guidelines for Reporting Reliability and Agreement Studies (GRRAS) statement.^26^ Prior to patient recruitment, a sample size calculation was performed using the G*Power software,^27^ version 3.1.9.7, which estimated a minimum sample size of 42 patient participants and five professional observers to achieve significant results with a large effect size, 95% power and a 95% confidence interval.

### Study setting and patient population

**Figure 2:**
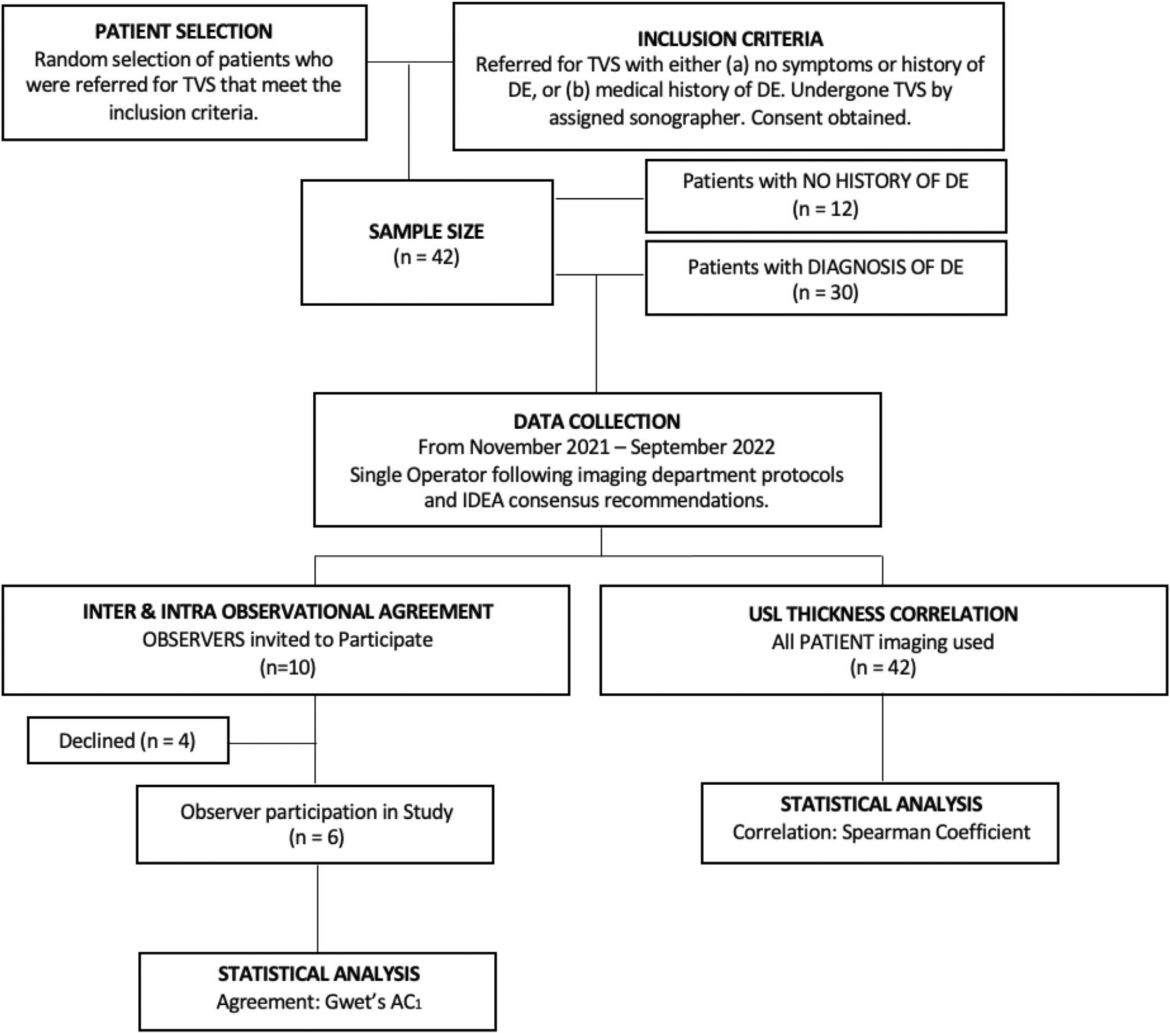
Flow chart summarising the methodology process and the inclusion criteria of eligible patients and observers in the study. *AC*_*1*_, agreement coefficient; DE, deep endometriosis; IDEA, international deep endometriosis analysis; n, sample size; TVS, transvaginal ultrasound; USL, uterosacral ligaments.

A total of 42 patient participants who met the inclusion criteria (Figure 2) were included in this study. These patients presented to an outpatient general imaging private practice in South Australia between November 2021 and September 2022, following referrals from general practitioners or gynaecologists. The 42 participants were divided into two groups: 30 with a known history of DE and 12 with no history or symptoms of DE. The 30 patients presenting with endometriosis had been medically diagnosed via either laparoscopy or clinically by a gynaecologist, and all exhibited symptoms suggestive of DE, such as dysmenorrhea, dyspareunia, chronic pelvic pain or infertility.^3^, ^4^

All 42 patient participants had a complete pelvic ultrasound, with TVS that was reported in line with clinic protocols for the referring physician. For each of the 42 patients, images and video clips evaluating the USLs were collated. The images and videos included in this study were selected based on representative diagnostic quality, obtaining a range of images from patients that displayed both normal and abnormal USLs to assess interobserver reliability.

### Study objectives

The objective was to find the inter‐ and intraobserver agreement in identifying ultrasound characteristics of DE in the USLs and ultrasound diagnosis of DE in the USLs across the six observers. This study also aimed to determine the correlation and agreement between USL DE and USL thickness on TVS.

### Data collection and image acquisition

Transvaginal ultrasound was performed by a highly skilled sonographer (SM) with 15 years of professional experience, using Canon i800, Aplio 500, Aplio a550 and Aplio XG (Canon Medical System Corporation, Otawara, Tochigi, Japan) ultrasound machines, equipped with 11CV3, 11CV or 9C3 (3–11 MHz) transvaginal transducers. Each examination included a complete pelvic ultrasound, covering the uterus, cervix, adnexa and ovaries, in accordance with general practice imaging protocols and the Australiasian Society of Ultrasound in Medicine (ASUM) guidelines^28^ for gynaecological ultrasound exams as per the referral request. The examination was then extended to assess for endometriosis, following the four‐step method outlined in the IDEA consensus,^9^, ^29^ which includes evaluation of the uterus, both adnexa, urinary bladder, vagina and rectovaginal septum, USLs and rectosigmoid. To ensure consistency and reproducibility, the recommended ultrasound techniques for visualising the USLs^4^, ^24^ were used. This involved placing the transducer in the posterior vaginal fornix, then withdrawing it into the vagina. The transducer was kept in the longitudinal plane and angled inferolaterally to visualise the cervix, before rotating it approximately 45° until the hyperechoic line of the USL was visible.^4^, ^24^ Measurements of the USLs were taken and recorded. Endometriosis was diagnosed based on the systematic approach described by the IDEA^9^ guidelines.

### Interobserver and intraobserver analysis

As noted previously, the sample size calculation indicated that five professional observers were needed to detect a large effect size. After invitations were extended, six observers agreed to participate, as shown in Figure 1. These included five accredited medical sonographers, as per the Australian Sonographers Accreditation Registry (ASAR), and one radiologist accredited by the Royal Australian and New Zealand College of Obstetricians and Gynaecologists (RANZCOG). This multidisciplinary approach, incorporating observers with varying levels of experience and qualifications, was designed to minimise professional bias and better reflect real‐world clinical practice.

Each observer reviewed the 42 cases in two separate sessions, approximately 8 weeks apart, to minimise recall bias. In each session, the same 42 cases were assessed, with each case including still images and video clips of the USLs for evaluation. The images were captured in the longitudinal plane, and the video clips provided a sweep through the posterior compartment, including sonopalpation to assess any potential adherence of the USL. Data and images for analysis were compiled for offline review using the Qualtrics software, version XM, June 2023, (Qualtrics, 2023, Provo, UT, USA). Observers were blinded to both the ultrasound outcomes and the responses of other observers, as well as to their own previous assessments.

Each of the six observers was asked to classify the ultrasound characteristics of each USL based on the current literature^24^ regarding how USLs should be described. This included evaluating echogenicity, echotexture, margin contour, thickness and the presence of any nodules. Observers were also asked to annotate in a text box where they would measure the USL, with responses categorised into five options: (i) Cervix/insertion, (ii) Thickest point, (iii) At nodule, (iv) Mid‐section or (v) Measurement not relevant. Although observers were not required to manually measure each USL, rather to answer subjectively, measurements were taken during the initial TVS examination of all patients, specifically at the thickest point and/or at any identified nodules and recorded for later analysis.

**Figure 3:**
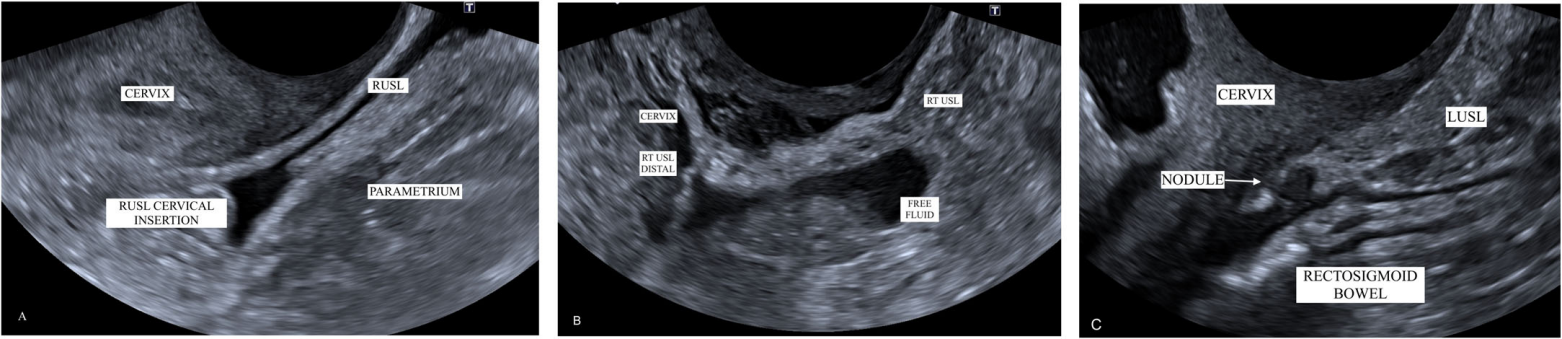
TVS ultrasound images of the USL. (a) a normal USL with a thin, hyperechoic, homogeneous, smooth structure with adjacent physiological fluid in a longitudinal plane. (b) USL with endometriotic involvement in a patient where the USL is highlighted with free fluid and seen to be thickened and heterogeneous with an irregular contour in a patient with endometriotic bowel lesions and background adenomyosis. (c) A hypoechoic endometriotic nodule within the USL at the cervical insertion. TVS, transvaginal ultrasound; USL, uterosacral ligaments.

The assessment criteria for the USLs were based on current review^24^ findings, which concluded that normal USLs appear as smooth, linear, hyperechoic and homogeneous structures that slide freely over the vaginal wall when transducer pressure is applied and released^24^ (Figure 3a). Uterosacral ligaments were considered to have endometriotic involvement if they appeared thickened with heterogeneous changes, or if there was hypoechoic linear thickening with regular or irregular margins (Figure 3b). Endometriotic nodules were identified as solid, hypoechoic nodules with regular, irregular or stellate margins, which were tender on sonopalpation and fixed to the surrounding pelvic structures^24^ (Figure 3c).

All USLs were measured longitudinally at the thickest section or at the level of any nodule. Observers were asked to subjectively assess whether the USL appeared thin or thickened, without being shown the recorded thickness values. Since there is no definitive standard measurement for the ultrasound diagnosis of USL DE,^24^ previous studies were used as a guide.^30^ Uterosacral ligaments were considered thickened if they measured greater than 3 mm,^31^ with a thickness greater than 5 mm^2^ indicating more significant disease involvement.

### Statistical analyses

**Figure 4:**
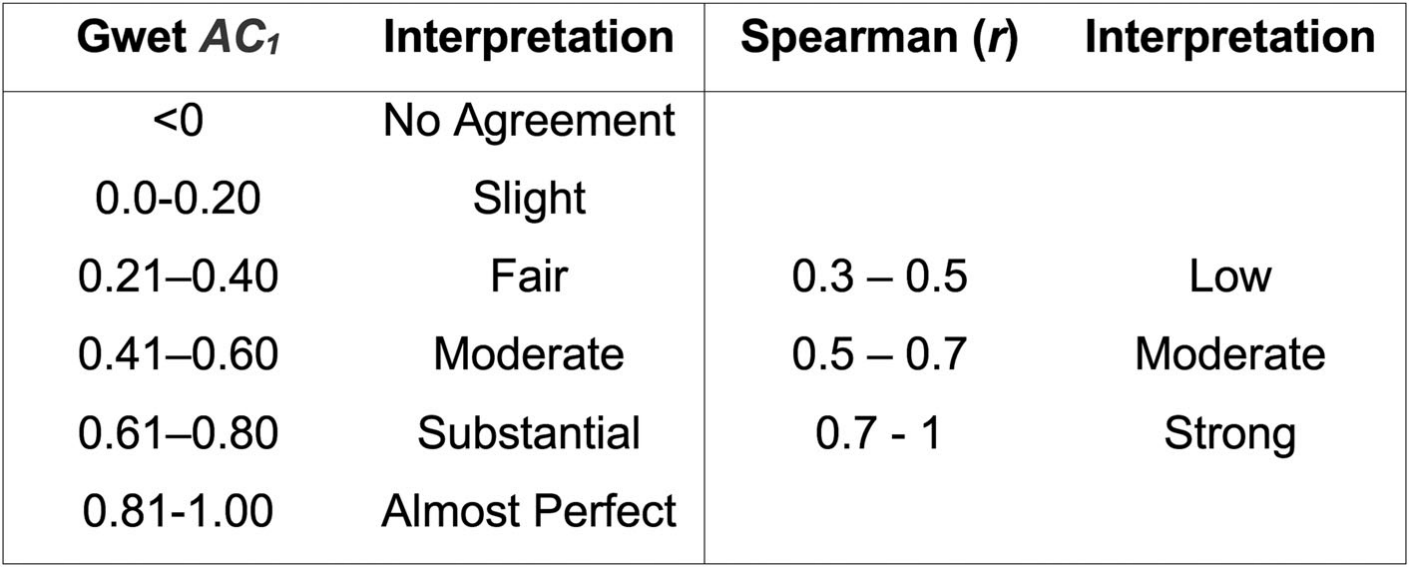
Scale used in statistics to quantify the level of agreement based Gwet *AC*_*1*_ values^32^ and Spearman coefficient (r) *AC*_*1*_ values.^32^ *AC*_*1*_, agreement coefficient.

The inter‐ and intraobserver reproducibility of TVS for diagnosing endometriosis in the USLs was assessed by calculating reliability and agreement using Gwet's agreement coefficients (*AC*_*1*_). Corresponding 95% confidence intervals (CIs) were computed to quantify interrater reliability, reflecting the consistency of observers in identifying the diagnostic characteristics of USLs indicative of endometriosis. Interobserver agreement, as interpreted through Gwet's AC_1_, was categorised as slight (0.0–0.20), fair (0.21–0.40), moderate (0.41–0.60), substantial (0.61–0.80) or almost perfect (0.81–1.00)^32^ (Figure 4). Statistical analyses were conducted using/SE 18.0 for Mac (Intel 64‐bit, revision 04 October 2023.^32^).

To assess the relationship between USL thickness and endometriosis, Spearman's correlation was performed using data from the 42 enrolled patients. The results were interpreted based on the strength of the correlation, with values categorised as strong (r = 0.7–1), moderate (r = 0.5–0.7) or low (r = 0.3–0.5), considering significant correlation values (P < 0.01 or P < 0.05) (Figure 4). A biostatistician was consulted to ensure the appropriateness of the analysis method.

### Ethics approval

The study was approved by the University of South Australia Human Research Ethics Committee (project number 204278), and written informed consent was obtained from all participants prior to inclusion in the study.

## Results

### Patient characteristics and ultrasound data sets

The characteristics of the patient participants and their presenting symptoms are summarised in Table 1. Forty‐two (42) female‐born patients were included, with a mean age of 33.6 years (range 18–54). Four patients over 50 years old, all with chronic endometriosis, were included in the final analysis. Ultrasound findings suggestive of endometriosis are outlined in Table 2.

**Table 1:** Patient characteristics, endometriosis history and indication for performing TVS of the study cohort (n = 42).

	(n = 42)	%
Patient characteristics		
Age	36 (Range 18–54) 33 (Median) 33.6 (Mean)	
No history DE	12	(28.6%)
DE diagnosed laparoscopy	10	(23.8%)
DE diagnosed clinically	7	(16.7%)
DE clinically investigated	13	(31%)
Indication for TVS		
Chronic pelvic pain	26	(61.9%)
Dysmenorrhea	16	(38.1%)
Infertility	6	(14.3%)
Dyspareunia	5	(11.9%)
Other	12	(29%)

DE, deep endometriosis; n, sample size; TVS, transvaginal ultrasound.

‘Other’ indications for TVS include: bloating, polycystic ovarian syndrome, follow‐up for endometrial polyp or hyperplasia, history of ovarian tumour, intrauterine contraception device location and unexplained weight loss.

**Table 2:** The findings of endometriosis on ultrasound for the patients with known or suspected history of endometriosis.

Findings of endometriosis on TVS		
	(n = 30)	%
*Participants with endometriosis Hx*	30	100
Endometriosis present	30	100
Endometrioma	14	46.7
Obliterated POD	4	13.3
Adherent ovaries	21	70.0
Medialised ovaries	9	30.0
Retroverted or rotated uterus	11	36.7
Adenomyosis	19	63.3
Total focal endometriotic lesions	21	70.0
Types of endometriotic lesions:		
Rectosigmoid DE	7	23.3
Rectocervical DE	9	30.0
USL DE (nodule)	12	40.0
Vaginal DE	0	0.0
Bladder DE	0	0.0
Uterine serosal plaques	2	6.7
LSCS scar endometriosis	1	3.3

DE, deep endometriosis; Hx, history; LSCS, lower section caesarean section; n, sample size; POD, pouch of douglas; TVS, transvaginal ultrasound; USL, uterosacral ligamen.

Detailed findings related to the USLs on TVS are summarised in Table 3. In patients with known DE, TVS revealed altered echotexture in all 30 USLs (100%), showing a more heterogeneous appearance compared with the homogeneous echotexture observed in participants without endometriosis. Specific ultrasound findings included 12 USL nodules (40%), 27 with irregular margins (90%), and all 30 USLs appearing thickened (100%).

**Table 3:** Ultrasound characteristics of the USLs on participants with and without history of endometriosis.

USL findings		
	No DE (n = 12)	DE (n = 30)
USL nodule	0	12 (40%)
Heterogeneous/Hypoechoic	0	30 (100%)
Irregular margins	0	27 (90%)
Thickened	0	30 (100%)
Thickness		
<3 mm	12 (100%)	0
3–5 mm	0	9 (30%)
>5 mm	0	21 (70%)

DE, deep endometriosis; mm, millimetres; n, sample size; USL, uterosacral ligaments.

No DE was detected in participants without a history or symptoms of DE. The USLs in these participants were normal and were recognised as such by the observers. However, it is important to address the absence of USL DE characteristics in asymptomatic or undiagnosed patients, particularly given that endometriosis can often be asymptomatic. This may suggest that the lack of detectable changes in the USLs of these patients does not necessarily rule out the presence of endometriosis, as the condition may remain undiagnosed or asymptomatic in many cases.^33^ To ensure accurate categorisation, the 12 participants without endometriosis were selected based on their lack of symptoms or indications of DE. These individuals underwent TVS for reasons unrelated to endometriosis, such as polycystic ovarian syndrome, follow‐up for endometrial polyps or hyperplasia, or intrauterine contraceptive device location (Table 1). In these cases, they were classified as normal.

Uterosacral ligament measurements varied across participants. In the 12 patients with no history of endometriosis, all had USLs measuring <3 mm. Among the 30 patients with known endometriosis, nine (30%) had USLs measuring 3‐5 mm, and 21 (70%) had USLs >5 mm, with the thickest measuring 9.2 mm.

### Interobserver agreement

According to Gwet AC_1_, the results show substantial overall agreement (0.65) for the seven ultrasound characteristics of the USLs across the six observers, confirming consistency and reproducibility. Table 4 demonstrates almost perfect agreement for echogenicity (0.96), substantial agreement for echotexture (0.63), presence of nodules (0.61) and nodule contour (0.63), as well as overall substantial agreement for normal versus abnormal USLs (0.63). Moderate agreement was observed for USL contour (0.42) and thickness (0.47), with fair agreement on the measurement site (0.29).

**Table 4:** Interobserver agreement and Gwet *AC*_*1*_ Statistics for ultrasound characteristics of USL across 6 observers.

Ultrasound USL characteristic (n = 42)	% Agreement	Gwet *AC*_*1*_ (95% CI)
Echogenicity	96	0.96 (0.92–0.99)
Echotexture	72	0.63 (0.51–0.75)
Contour of USL	71	0.42 (0.25–0.59)
Nodule present	76	0.61 (0.44–0.78)
Contour of nodule	69	0.63 (0.50–0.76)
Thickness	73	0.47 (0.31–0.63)
No Hx DE (n = 12)	88	0.86 (0.66–1.0)
Hx DE (n = 30)	68	0.42 (0.21–0.63)
Measurement site	39	0.29 (0.24–0.33)
Normal v Abnormal	72	0.63 (0.53–0.74)
Overall	71	0.65 (0.62–0.69)

AC_1_, agreement coefficient; CI, confidence interval; DE, deep endometriosis; Hx, history; n, sample size; USL, uterosacral ligaments.

Table 5 shows substantial agreement (0.63) for the ultrasound diagnosis of endometriosis in the USLs across all six observers. Notably, there was a substantial agreement for patients without a clinical diagnosis of DE (0.66) compared with moderate agreement (0.56) for those with known DE.

**Table 5:** Interobserver agreement and Gwet *AC*_*1*_ Statistics for ultrasound diagnosis based on ultrasound findings of endometriosis of the USL across 6 observers.

	% Agreement	Gwet *AC*_*1*_ (95% CI)
Overall	72	0.63 (0.53–0.74)
Patients with no clinical DE	75	0.66 (0.39–0.93)
Patients with known DE	72	0.56 (0.36–0.76)

AC_1_, agreement coefficient; CI, confidence interval; USL, uterosacral ligaments.

### Intraobserver agreement

As shown in Table 6, all six observers demonstrated high intraobserver agreement, with values ranging from moderate (0.60) to almost perfect (0.96). One observer had a moderate agreement (0.60), which was just 0.01 below the threshold for substantial agreement. Three observers had substantial agreements (0.74, 0.76 and 0.80), and two had almost perfect agreements (0.87 and 0.96). These results also indicated that years of experience did not significantly influence intraobserver reliability.

**Table 6:** Intraobserver agreement and Gwet *AC*_*1*_ Statistics for ultrasound characteristics of USL across 6 observers.

	Years of experience	% Agreement	Gwet *AC*_*1*_ (95% CI)
Observer A	14	84	0.80 (0.78–0.90)
Observer B	7	89	0.87 (0.81–0.92)
Observer C	2	79	0.76 (0.69–0.82)
Observer D	4.5	78	0.74 (0.67–0.81)
Observer E	11	69	0.60 (0.52–0.68)
Observer F	16	96	0.96 (0.93–0.99)

AC_1_, agreement coefficient; CI, confidence interval; USL, uterosacral ligaments.

### USL measurement agreement and correlation

Moderate interobserver agreement (0.47) was observed for USL thickness (Table 4). For patients with no DE, there was almost perfect agreement (0.86) in identifying thin USLs, while for patients with DE, the agreement dropped to moderate (0.42). Discrepancy also existed in determining where to measure the USL, with a fair agreement (0.29). As shown in Table 7, 92% of observers correctly identified the USL as thin in patients with no history of DE, but there was more variation in identifying thickened USLs in patients with DE (69%).

**Table 7:** Percentage of ‘thin’ and ‘thick’ rated USLs across six observers.

	Patients with no clinical DE (n = 12)		Patients with known DE (n = 30)	
	Thin	% identified thin for normal	Thick	% identified thick for abnormal
Observer A	12/12	100	11/30	36
Observer B	11/12	92	22/30	73
Observer C	9/12	75	29/30	97
Observer D	11/12	92	16/30	73
Observer E	11/12	92	22/30	73
Observer F	12/12	100	25/30	83
Overall	66/72	92	125/180	69

DE, deep endometriosis; n, sample size; USL, uterosacral ligaments.

**Figure 5:**
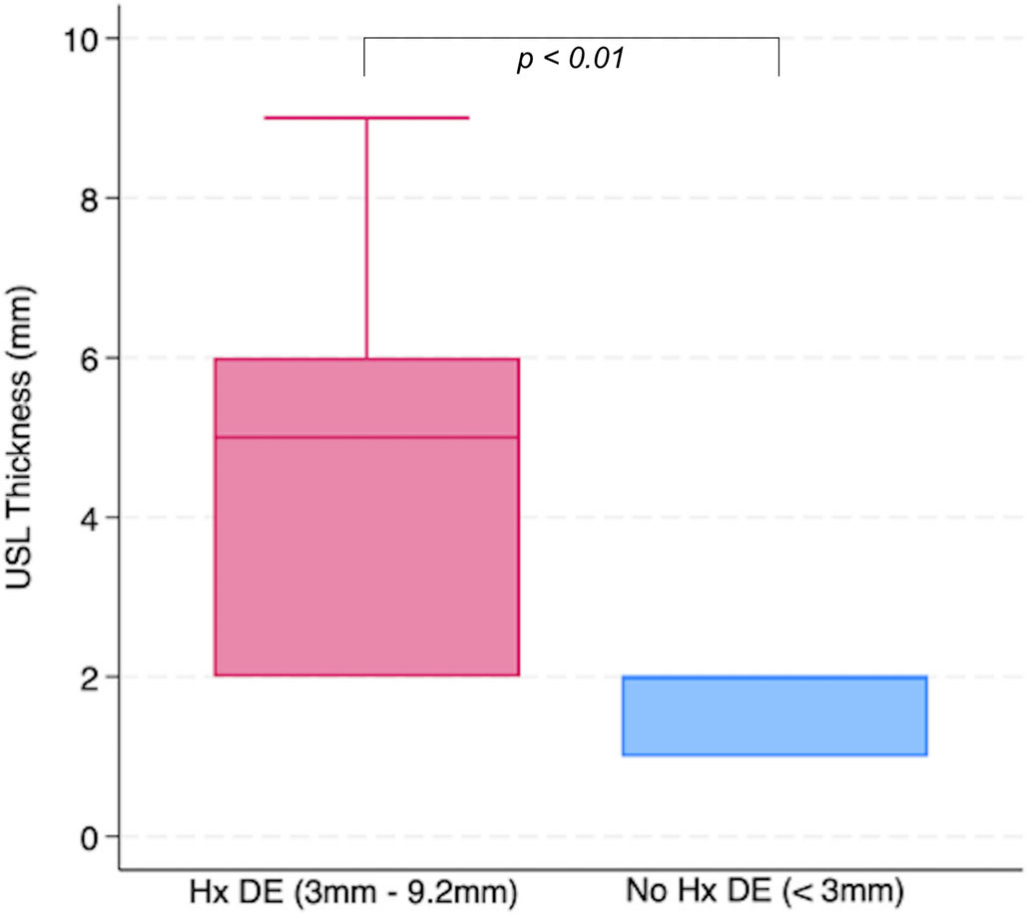
Box plot demonstrating a strong correlation of USL thickness vs. patient endometriosis history. DE, deep endometriosis; Hx, history; mm, millimetres; USL, uterosacral ligaments.

The correlation between USL thickness and the presence of endometriosis showed a strong positive relationship, which was statistically significant (r = 0.7965, P < 0.01), as illustrated in the box plot (Figure 5).

## Discussion

### Inter‐ and intraobservational agreement

The results of this study have addressed the objectives and demonstrated high intra‐ and interobservational reproducibility of TVS in assessing the ultrasound characteristics of USLs for diagnosing DE. These findings align with the current systematic review,^24^ which, although unvalidated, also reported good consensus on USL characteristics in the literature.

The interobserver agreement for ultrasound diagnosis of endometriosis showed substantial agreement (0.63), with higher agreement for identifying normal USLs (0.66) compared with abnormal USLs (0.56). This suggests that observers had a clear understanding of normal USL appearance but showed some variability in identifying abnormal features. For instance, there was almost perfect agreement on echogenicity (0.96), indicating identifiable diagnostic criteria for assessing USLs. However, there was only moderate reliability for USL contour and thickness, which aligns with previous studies suggesting that USL thickness has poor sensitivity for detecting endometriosis.^30^ This highlights the need for further education in this area.

**Figure 6:**
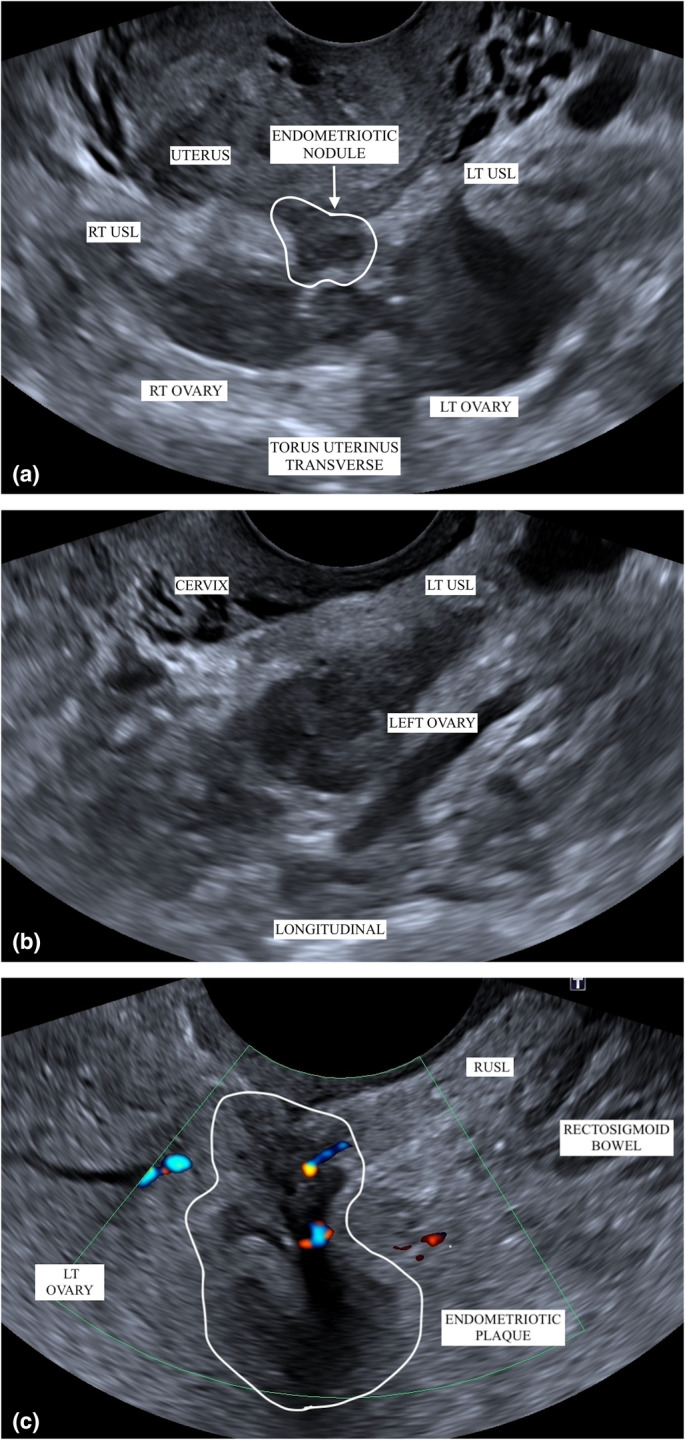
Laparoscopy diagnosed endometriosis with TVS appearances of DE. (a) Transverse plane of bilateral ovarian endometrioma with medialisation of both ovaries adherent to the USLs and the uterine fundus. There is the appearance of an endometriotic nodule at the TU involving both USLs. (b) The LUSLs demonstrated longitudinally is seen thickened, heterogeneous and irregular with the left ovaried adhered. (c) Frozen pelvis, with extensive deep infiltrative endometriosis where the patient report focal sharp pain during sonopalpation. There is endometriosis plaque around the level of the rectosigmoid bowel with appears tethered to the inferior margin of the ovaries, cervix and extends to the right USL with tethering. DE, deep endometriosis; TU, torus uterinus; TVS, transvaginal ultrasound; USL, uterosacral ligaments.

Interobserver agreement on the presence of nodules demonstrated substantial agreement (0.61), indicating that, even with limited criteria, observers can reliably identify DE nodules in the USLs and posterior pelvic compartment. This ability aids both diagnosis and treatment planning, contributing to better patient outcomes, particularly since nodules at the torus uterinus (TU) are frequently affected, with 95.7% of bilateral USL DE cases showing TU plaques.^15^ While DE nodules typically occur in the cervical aspect of the USL near the TU insertion^21^ (Figure 6a), they can also affect multiple anatomical sites^24^ (Figure 6b) and may infiltrate the parametrium^34^, ^35^ (Figure 6c). This infiltration is often associated with ovarian adhesion, a common cause of medialised ovaries, especially in the presence of endometriomas^21^ (Figure 6a,b), contributing to higher symptomatic pain, as well as tenderness during TVS examination.^36^

Correctly identifying disease extension, such as infiltration into adjacent structures, is essential for planning more complex surgery.^13^, ^35^ Endometriosis in these areas can distort anatomy, particularly when the pouch of Douglas (POD) is obliterated,^4^ and increase the risk of ureteral involvement.^35^ Furthermore, isolated USL endometriotic lesions are common in patients under the age of 25 with severe dysmenorrhea and are often overlooked at laparoscopy.^37^ This underscores the importance of pre‐surgical planning, as excision of USL DE often requires advanced laparoscopic skills, especially in cases involving an obliterated POD.^4^, ^13^ Meaning, sonographer application of the findings of this study may have important clinical implications.

Intraobserver results showed strong agreement, with two observers achieving almost perfect agreement and three others showing substantial agreement. These findings further support the idea that USL characteristics change with DE infiltration and that thickening of the USL is suggestive of endometriosis. These changes can be consistently identified by trained professionals across various disciplines and experience levels, provided the criteria are clear. Once reproducible guidelines and departmental protocols are established, along with targeted sonographer education, further research is needed in general settings to assess whether this approach can improve diagnostic accuracy and facilitate earlier diagnosis.

### Measurement correlation

Although taking measurements of the USLs is recommended by the IDEA^9^ consensus, the normal thickness of USLs is currently unknown and the standard anatomic plane for reproducible measurements for USLs is not validated.^24^ This study found that the subjective thickness for taking measurements of the USLs were the most ambiguous with observers’ identification of the thickness of the USL most difficult, with moderate interobserver agreement (0.47), particularly for patients with a history of DE. There was further discrepancy in agreement of where to measure the USL with a fair (0.29) agreement. The observers were asked to annotate freely in a text box as to where they would measure the USL. Generally, the consensus was to measure at the cervical insertion if the USL appeared normal, and to measure the thickest part or at the nodule if the USL was showing signs of DE although agreement was inconsistent.

Patient data, as seen on Table 3, highlighted those patients who had no evidence of DE had USLs all <3 mm, while those patients with known DE had USL measurements ranging from 3.4 mm to 9.2 mm (mean 4.5 mm). Observers identified USLs <3 mm as thin 92% of the time and >3 mm as thickened 69% of the time (Table 7). This indicates that USL thickness can serve as a subjective marker for DE. The strong correlation between USL thickness and DE further supports its potential clinical value for early detection, provided reproducible and validated measurement criteria are established.

## Strengths, limitations and future studies

This study is the first to assess the reproducibility of ultrasound criteria for USLs in the diagnosis of endometriosis, testing the applicability of the IDEA^9^ consensus and findings from systematic reviews^24^ in general, non‐specialist clinical settings. While previous studies have examined interobserver variability in ultrasound diagnosis of endometriosis, they have all been conducted in tertiary, specialist settings.^14^, ^38^

Strengths of this study include achieving the required sample size for a well‐powered analysis, a prospective design, examiner blinding, a multidisciplinary approach and the fact that all assessments were performed at a single centre. Images were collected by one sonographer using real‐time ultrasound, and scans were conducted following a rigorous best‐practice protocol, further strengthening the study's reliability.

A notable advantage of this study is that observers were not exposed to non‐USL pelvic structures, thereby minimising potential confounders, such as endometriomas, during TVS assessments. However, a limitation lies in the lack of blinding for the sonographer performing the scans, as they were aware of other pelvic pathologies, introducing the risk of confirmation bias. Ideally, future studies aiming to evaluate USL findings should isolate their assessment, independent of broader pelvic or endometriosis evaluations, to eliminate such bias.

In addition to this, further limitations must be considered. The study focused solely on TVS appearances of USLs, and for patients who underwent laparoscopic excision of endometriosis, it is unknown whether USL excision was performed. Given that the USL is a common site for endometriosis and diagnostic surgeries, it is likely that some cases involved surgical intervention, potentially altering USL appearance, which may have impacted the sonographic findings. This represents a significant limitation, as there is uncertainty around whether all measured USLs were from native tissue or if they had been altered post‐surgically. Furthermore, it is unclear which USLs may have changed in appearance due to surgery, as this information was not available, thus making it difficult to interpret whether these changes affected the results. Additionally, the study included six observers to assess interrater agreement, and the use of offline images and videos may introduce bias related to the relevance of operator experience. Future studies should involve both novices and experts performing the scans after receiving clear criteria, education and training, and then compare the performance of novices with that of experts to assess the feasibility and effectiveness of this approach. However, ethical and logistical considerations may pose challenges to implementing this design.

Despite these limitations, this study demonstrates significant interobserver reliability, potentially laying the groundwork for future studies that validate the findings discussed in this study including TVS diagnostic characteristics of USL DE, improved understanding of normal and abnormal USLs. Further research is needed to establish reliable cut‐offs for USL thickness and to determine its clinical relevance. Additionally, studies should explore the impact of implementing the IDEA^9^ consensus and rigorous scanning protocols in general clinical settings, as well as the need for updated and standardised scanning protocols in general ultrasound departments. Lastly, further investigation is required to assess the reproducibility of TVS USL examination in general settings and whether additional training is necessary for sonographers and radiologists.

## Conclusion

This study demonstrates high inter‐ and intraobserver agreement among sonographers and imaging professionals in a general imaging department for identifying ultrasound characteristics of USLs and diagnosing DE using TVS, when current recommended guidelines are followed. It confirms that TVS is a reliable tool across both experienced and less experienced examiners in general imaging settings and shows that trained professionals can consistently identify key USL characteristics, including echogenicity, thickness and nodules, thereby supporting the use of TVS in general imaging for detecting USL DE.

Notably, there was strong agreement in identifying abnormal USLs, particularly in detecting nodules, while agreement on USL thickness and measurement site was more variable. The study also found a strong positive correlation between USL thickness and the presence of DE. However, there is no consensus on the optimal site for measuring the USL. Despite this, incorporating USL examination into routine pelvic scans may be helpful for detecting thickening, which could prompt further investigation for other endometriosis markers. This approach is valuable especially in general imaging settings, where skill levels may vary, as identifying these markers can help facilitate referral to a specialist centre, as recommended by the IDEA^9^ consensus.

While reproducible measurement criteria and sonographer education are necessary, the results suggest that TVS, when applied with clear criteria, could improve early diagnosis and treatment planning for endometriosis. This research contributes to the growing body of evidence supporting the clinical value of TVS in endometriosis management, offering insights into how sonographers can play a crucial role in early detection and improved patient outcomes.

## Author Contributions

**Shae Maple:** Conceptualization; methodology; validation; formal analysis; investigation; resources; data curation; writing – original draft preparation; writing – review and editing; supervision; project administration. **Eva Bezak:** Conceptualization; methodology; validation; formal analysis; investigation; writing – review and editing; supervision; project administration. **K Jane Chalmers:** Conceptualization; methodology; supervision; project administration. **Nayana Parange:** Conceptualization; methodology; validation; formal analysis; investigation; writing – review and editing; supervision; project administration. All authors have read and agreed to the published version of the manuscript.

## Funding

This research received no external funding.

## Conflict of interest

The authors declare no conflict of interest.

## Disclosure statement

Nayana Parange is an associate editor for AJUM. This research is supported by The University of South Australia.

## Data Availability

The data presented in this study are available upon request from the corresponding author due to patient confidentiality restrictions.
